# License plate recognition methodology in complex scenarios based on CSCM-YOLOv8 and CSM-LPRNet

**DOI:** 10.1371/journal.pone.0339649

**Published:** 2026-01-02

**Authors:** Weihua Xiong, Lixian Cao, Dongming Yan, Yufei Jiang, Guan Zhang, Yan Wang, Xiaotong Huang, Liquan Liu

**Affiliations:** 1 School of Information and Control Engineering, Jilin University of Chemical Technology, Jilin, China; 2 School of Optoelectronic Engineering, Guilin University of Electronic Technology, Guilin, Guangxi Zhuang Autonomous Region, China; Macau University of Science and Technology, MACAO

## Abstract

License plate recognition technology is widely applied traffic management, parking monitoring, and electronic toll collection, among other fields. However, in complex scenarios, such as bright light, fog, rain, snow, and nighttime, there is an urgent need to improve the accuracy of license plate recognition and system robustness. To cope with the difficult problem of license plate recognition in complex scenarios, this study proposes a license plate recognition method based on CSCM-YOLOv8 and CSM-LPRNet. The CPA-Enhancer preprocessing module is used to optimize the input feature representation, and the upsampling quality is improved by the perceptual feature reorganization capability of the CARAFE upsampling module. The SEAM is embedded for adaptive weight allocation, thus enhancing the capability to extract key features. The SEAM is combined with the lightweight C2fMLLABlock convolution module to efficiently aggregate features, thereby maintaining the feature representation capability while reducing the computational cost. The experimental results show that on the dataset used in this study, the CSCM-YOLOv8 network achieves 98.9% accuracy in license plate detection, whereas mAP@0.50-0.95 reaches 58.0%. Compared with the original YOLOv8, the accuracy and mAP@0.50-0.95 are improved by 3.1% and 3.9%, respectively. Moreover, CSM-LPRNet achieves a recognition accuracy of 98.56% in character recognition, which is a 7.0% improvement over that of the original LPRNet. The remarkable performance of this method in complex environments provides an efficient and reliable solution for license plate recognition in intelligent transportation systems.

## 1 Introduction

License plate recognition technology [1] plays a key role in areas such as traffic management, security monitoring, and smart city development. By leveraging this technology, vehicles can be identified quickly and accurately to improve traffic efficiency and safety. In urban transportation, license plate recognition can support violation monitoring, automatic toll collection, and parking management to reduce human labor and increase management efficiency. In addition, within intelligent transportation systems, license plate recognition provides a foundation for data analysis to optimize traffic flow distribution and reduce congestion.

With the advancement of technology, the scope and accuracy of license plate recognition will continue to improve and bring greater convenience and safety to society. Despite this progress, the technology still faces challenges in practical applications due to complex environments [2]. Traditional methods often perform poorly in degraded scenarios, such as low light, rain, and snow, making it difficult to meet real-time and accuracy requirements. A typical license plate recognition system usually consists of three key steps: license plate image preprocessing, license plate detection, and character recognition [3]. Image preprocessing is intended to enhance image quality and provide clearer input for subsequent detection. Existing methods, such as grayscaling [4], histogram equalization [5], filter denoising [6], and geometric correction techniques [7],help improve image quality but remain less effective for low-light or noise-disturbed images. License plate detection is a critical component of license plate recognition, and it aims to accurately locate the license plate region within complex backgrounds. However, traditional rule-based or feature-engineering methods, such as color thresholding for image segmentation [8], rectangular shape detection [9], Haar feature [10], and HOG feature sliding window detection [11], show reduced detection accuracy under conditions such as changing illumination and occluded plates. These approaches rely on handcrafted features with limited generalization ability, making them unsuitable for diverse license plate types. Character recognition is the final step of the system, and it aims to extract and recognize characters from the detected license plate region. Traditional character recognition methods often adopt a step-by-step strategy for character segmentation and classification [12], but their accuracy decreases sharply when processing connected characters, blurred fonts, or strong background interference.

In recent years, the rapid advancement of deep learning has brought new methods to improve the performance of license plate recognition systems. Target detection and character recognition methods based on convolutional neural networks (CNNs [13] have gradually become mainstream. For example, Wang et al. [14] achieved real-time defect detection of metal components by integrating an enhanced Canny-Devernay algorithm with YOLOv6, demonstrating the strong effectiveness of the YOLO framework in complex industrial environments. The YOLO [15] series models have demonstrated excellent efficiency and accuracy in license plate detection tasks owing to their end-to-end architecture for detection. The two-stage ALPR framework proposed by Ke et al. [16] achieves high efficiency in license plate detection and recognition by improving YOLOv3-tiny and lightweight MRNet. However, limitations exist in terms of adaptability in extreme environments and dependence on data enhancement. The system based on YOLOv7 and LPRNet proposed by Pan et al. [17] shows strong robustness in Chinese license plate recognition tasks. However, its performance is insufficient when dealing with occlusion, lighting changes, and complex backgrounds. In addition, Moussaoui et al. [18] proposed a recognition method based on YOLOv8 and OCR that improves the visibility of characters through image enhancement. However, the problem of OCR recognition rate degradation when dealing with blurred and concatenated characters has not yet been effectively addressed. These studies show that although existing deep learning techniques provide efficient solutions for license plate recognition, their adaptability in extreme environments and robustness to complex scenarios still need to be further improved.

However, pure CNN models have inherent limitations in modeling long-range dependencies and capturing global contextual information, which restricts their performance under severe occlusion, extreme lighting conditions, and complex background interference. To address this limitation and draw inspiration from advanced perception methods developed for unstructured environments in other fields, such as the geometry-aware 3D point cloud learning approach proposed by Wang et al. [19] for precise cutting point detection in unstructured field environments, whose central idea strengthens geometric structure perception through deep networks, Visual Transformer models and their variants have been introduced into computer vision. Liu et al. [20] proposed the Swin Transformer, which constructs hierarchical feature maps through a shifted window mechanism, effectively reducing computational cost and improving global context modeling, enabling rapid adoption as a strong vision backbone network. Building on this progress, Transformer-based solutions have emerged in license plate recognition. Azadbakht et al. [21] directly applied the Vision Transformer architecture for sequence recognition and introduced the Vision-LPR model, which improves recognition performance for blurred and low-resolution license plates by leveraging global attention mechanisms. Extending this direction, Dittakan et al. [22] developed an end-to-end Transformer-based license plate recognition framework named LPSTR, integrating detection and recognition in a single model and achieving state-of-the-art performance on several challenging benchmark datasets.

Despite the strong potential demonstrated by advanced Transformer-based methods, they also encounter challenges such as model complexity, high computational cost, relatively slow inference speed, and heavy dependence on large training datasets [23]. These limitations restrict their use in resource-constrained environments or applications that require strict real-time performance.

In summary, current research shows that CNN models such as YOLOv8 still offer irreplaceable advantages in efficiency and deployment convenience, while Transformer architectures provide strong performance but remain limited by computational overhead and uncertain generalization ability. Therefore, this paper builds on YOLOv8 [24] which reaches a balanced trade-off between efficiency and performance, by combining the CPA-Enhancer [25], SEAM [26], Content-Aware ReAssembly of FEatures (CARAFE) [27] and MLLABlock [28] models. This study also introduces the SE [29], CBAM [30] and MixConv2d [31] models into the LPRNet [32] to construct a CSM-LPRNet-based character recognition method that improves recognition ability in complex environments.

## 2 Problem analysis

**Fig 1:**
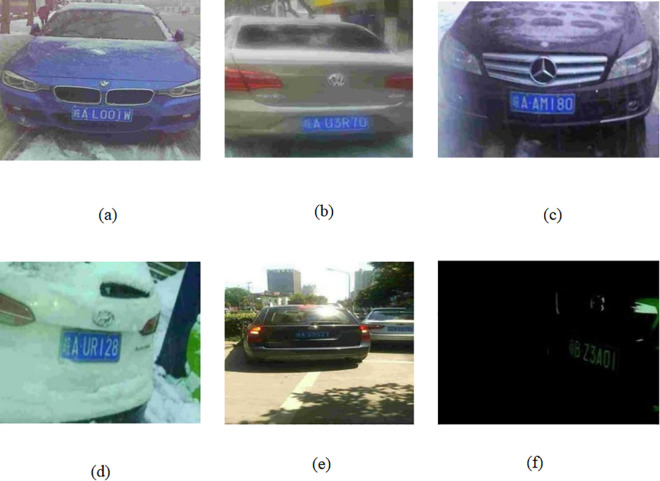
License plate images in complex scenarios.

Fig 1 shows license plate images under complex scenarios [33]. In some conditions, such as fog, the regions surrounding the license plate become blurred, and the overall contrast decreases, causing the edge contours to appear unclear, as shown in Fig 1(a) and (b). In heavy rainfall, raindrops on the surface of the plate may reflect light and introduce blurring effects, and some characters may be partially obscured by droplets, reducing clarity and contrast, as shown in Fig 1(c). In heavy snowfall, characters may appear incomplete because of partial or full occlusion, and strong ambient light or reflections may further reduce plate contrast, as illustrated in Fig 1(d). Under bright light conditions, the plate surface may show overexposure due to strong illumination or reflection, causing some characters to become blurred or difficult to recognize, as shown in Fig 1(e). In low-light or no-light environments, limited illumination can cause the characters on the license plate to become blurred or even invisible when the surrounding background is dim, as shown in Fig 1(f).

**Fig 2:**
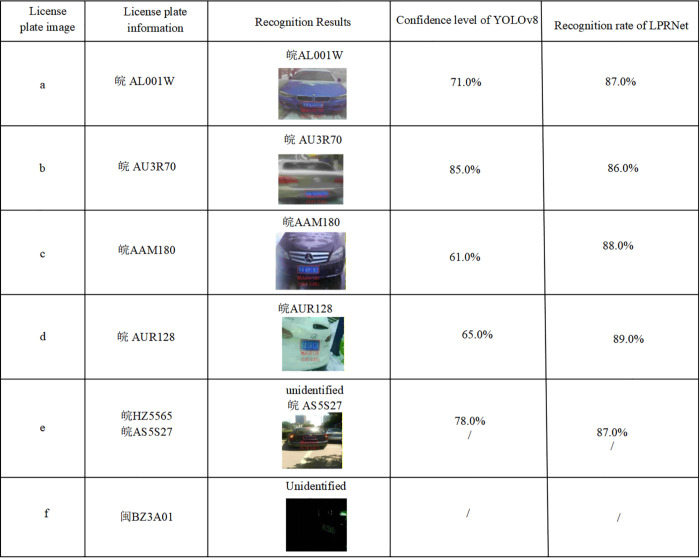
License plate recognition performance analysis of the YOLOv8 and LPRNet methods.

In complex scenarios, when YOLOv8 is used for license plate detection, and LPRNet is used for character recognition, factors such as fog, raindrops, and snow often blur the license plate region or obscure characters, resulting in less accurate localization and lower output confidence values [34]. In addition, strong light reflection and low-light conditions [35] further reduce character-edge clarity, leading to insufficient feature extraction by LPRNet, which lowers the recognition rate and affects overall character recognition accuracy. As shown in Fig 2, the impact of complex scenarios on detection and recognition performance is particularly evident.

## 3 Methodology

### 3.1 Overall architecture

**Fig 3:**
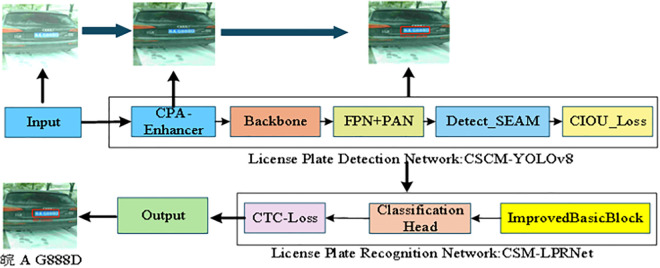
License plate recognition system flowchart.

Fig 3 shows the license plate recognition system’s workflow. The system consists of two main components: license plate detection and character recognition.

During the detection phase, the system uses the CSCM-YOLOv8 model to localize and detect license plates. This model includes the CPA-Enhancer module, the Backbone network, the FPN+PAN structure, the Detect-Separated and Enhancement Attention Module (SEAM) module, and the CIOU_Loss module. The input image is first processed by the CPA-Enhancer to improve contrast and detail, increasing robustness under complex lighting conditions. The enhanced image is then sent to the Backbone network for multi-level feature extraction. The resulting high-level semantic features are passed to the FPN+PAN structure, where a bidirectional top-down and bottom-up pathway enables effective fusion of shallow and deep features, improving small-object detection and localization accuracy. After feature fusion, the Detect-SEAM attention module recalibrates both spatial and channel features, increasing sensitivity to small license plates. Finally, the CIoU loss function is used as the optimization objective for bounding box regression, considering the overlap area, center distance, and aspect ratio to achieve more accurate detection box prediction.

In the recognition stage, the license plate regions localized and cropped by the CSCM-YOLOv8 model are passed to the CSM-LPRNet character recognition network for end-to-end sequence recognition. This network uses the BasicBlock as its fundamental unit, applying residual connections to improve gradient propagation and reduce performance degradation during deep network training. The Classification Head maps high-level features to probability distributions over character classes. To address alignment challenges in sequence prediction, the model adopts the CTC loss function, which enables stable recognition of variable-length license plate numbers without requiring character-level pre-segmentation.

Finally,the system outputs the complete character information from the license plate, achieving an efficient recognition pipeline from input image to license plate text.

### 3.2 CSCM-YOLOv8 model

**Fig 4:**
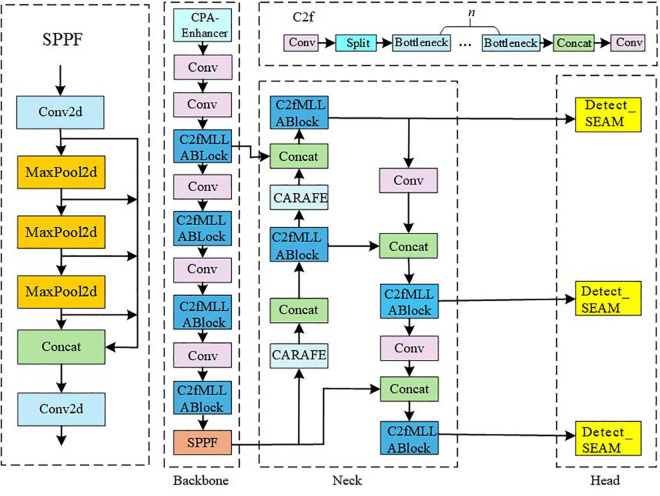
CSCM-YOLOv8 model diagram.

Given the complexity of the license plate detection task, the model must exhibit strong anti-interference ability and accurately detect small targets and partially occluded objects. Therefore, this study proposes the CSCM-YOLOv8 model, as shown in Fig 4, to address detection challenges in complex scenarios. The CPA-Enhancer module [36] enhances the backbone network’s ability to capture multiscale features by providing richer feature representations. Combined with the CARAFE [27] adaptive downsampling method, the proposed model reduces information loss and improves detection accuracy for multi-scale targets. The introduction of the C2fMLLABlock [28] strengthens feature expression and improves computational efficiency by reducing redundant operations. In addition, the SEAM [37] module enhances feature-map representation, enabling the model to focus on key regions and further improving the accuracy and robustness of license plate detection.

#### 3.2.1 CPA-enhancer module.

**Fig 5:**
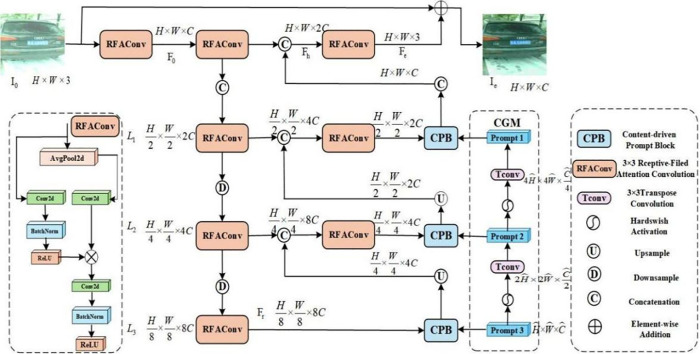
CPA-Enhancer model architecture.

License plate images are often affected by illumination variations, occlusion, and motion blur, which lead to considerable differences in extracted features. Therefore, this study introduces the CPA-Enhancer module [38] before the original backbone network of YOLOv8. The module, guided by chain-of-thought (CoT) hints, can dynamically adjust the enhancement strategy to adapt to the different features of an image. The CPA-Enhancer functions as an independent front-end network. It receives the original RGB image as input and produces an enhanced feature map as output. This enhanced feature map then replaces the original image as the input to the YOLOv8 backbone network. The CPA-Enhancer module consists of multiple enhancement sub-modules, each focused on optimizing specific image features to achieve layer-by-layer fine extraction and processing. Its chain-like design fully leverages the guiding role of CoT, enhancing the representation capability of image features through step-by-step reasoning. Fig 5 shows the specific architecture of the CPA-Enhancer.

First, the initial input license plate is *I*_0_(H×W×3), and features of *I*_0_ are extracted using receptive-field attention convolution (RFAConv) to obtain the initial features *F*_0_,$F_{0}=\text{RFACov}(I_{0})$. The dimensions remain as H×W×C forming the basis for feature extraction in the subsequent encoder.

Then, *F*_0_ is fed into an RFAConv layer to extract deeper contextual information and generate a high-level feature map *F*_*h*_.

Here, $F_{h}=\text{RFACov}(F_{0})$. The size of the feature map has a size of H×W×2C. In addition, *F*_0_ and *F*_*h*_ are fused to produce the final enhanced feature map with a size of H×W×3.

The feature *L*_1_ is extracted from the first layer, and *F*_*e*_ extracts high-resolution featuresthrough concatenation and RFAConv, thus, $L_{1}=RFAConv(Concat(F_{0},F_{h})),\;L_{1}\in {\mathbb{R}}^{H/2\times W/2\times 2C}$.

Moreover, *L*_2_ is the is the feature extracted from the second layer. It is generated by downsampling *L*_1_ and extracting the medium resolution features using RFAConv, thus yielding $L_{2}=RFAConv(Downsample(L_{1}))),\;L_{2}\in {\mathbb{R}}^{H/4\times W/4\times 4C}$.

Furthermore, *L*_3_is the feature from the third layer. It is generated by downsampling *L*_2_ and extracting low-resolution features using RFAConv, thus:$L_{1}=RFAConv(Downsample(L_{1}))),\;L_{3}\in {\mathbb{R}}^{H/8\times W/8\times 8C}$.

Deep features *F*_*r*_ are extracted by applying RFAConv to *L*_3_, producing$F_{r}=RFAConv(L_{3})),\;F_{r}\in {\mathbb{R}}^{H/8\times W/8\times 8C}$.

**Fig 6:**
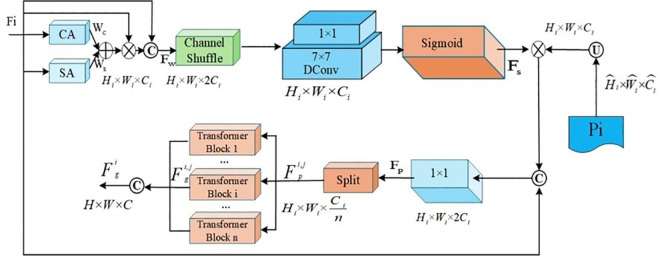
The structure of the CPB module.

The multiscale features *L*_1_,*L*_2_,*L*_3_,*F*_*r*_ are then fed into the CPB module for optimization. As shown in Fig 6, in the CPB module, the input feature $F_{i}\in \{L_{1},L_{2},L_{3},F_{r}\}$ interacts with the prompt *P*_*i*_ to compute the spatial and channel importance maps*A*_*s*_ , $S_{i}=\text{Attetion}(F_{i})$ ,$M_{i}=S_{i}\;\cdot \;F_{i}$ through the attention mechanism. Next, *M*_*i*_is split into *n* parts along the channel dimensions, and these parts are processed through independent Transformer blocks separately for feature optimization:$M_{i}^{j}=\text{Transformer}(M_{i}^{j},P_{i}),j=1,2,\ldots ,n$. All segmented parts are concatenated along the channel dimension to generate the optimized feature maps,$F_{i}^{cpb}=\text{Concat}(M_{i}^{1^{\prime }},M_{i}^{2^{\prime }},\cdots ,M_{i}^{n^{\prime }})$.

In the CGM module, the multiscale prompts are generated from $F_{i}^{cpb}$ using transposed convolution, $P_{i}=Tconv(F_{i}^{cpb})$. Hard Swish activation controls the information flow to ensure effective feature transfer. Here,$P_{i}=Hardwish(P_{i}))$), the low-resolution prompts are upsampled, and the high-resolution prompts are aligned: $P_{2}^{up}=\text{Tconv}(P_{2})$,$P_{3}^{up}=\text{Tconv}(P_{3})$. The high-, medium-, and low-resolution prompts are then fused to generate the final enhanced feature map *I*_*e*_, where $I_{e}=\text{Concat}((P_{1}),(P_{2}^{up}),(P_{3}^{up}))$.

Finally, the enhanced feature map after optimization and fusion *I*_*e*_ is fed into the target detector to generate the detection results: $textResuts=\text{Detector}(I_{e})$.

This study adopts a two-stage training strategy. In the first stage, the pre-trained YOLOv8 backbone network is frozen, and only the CPA-Enhancer module is trained with a learning rate of $1\;\times \;10^{-3}$ to allow rapid adaptation to the feature enhancement task. In the second stage, the entire network is unfrozen for end-to-end joint fine-tuning with a learning rate of $5\;\times \;10^{-4}$, utilizing a cosine annealing scheduler. All experiments are conducted using the PyTorch framework with the AdamW optimizer, and input images are uniformly resized to 640×640 pixels.

#### 3.2.2 CARAFE module.

**Fig 7:**
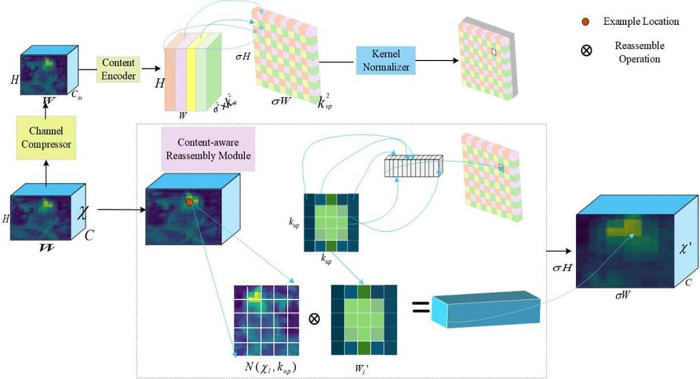
CARAFE module diagram.

The YOLOv8 upsampling stage uses nearest-neighbor interpolation. However, in license plate recognition, character strokes and plate borders are small yet critical features, and losing these details during upsampling can severely affect detection accuracy. Although nearest-neighbor interpolation is computationally efficient, it often produces blurred edges and fails to reconstruct fine character and contour structures, weakening the model’s ability to perceive key features and reducing recognition accuracy. To address this limitation, this study introduces the CARAFE module [39]. The CARAFE module recovers fine details through a content-aware mechanism that significantly enhances detail restoration and feature representation in complex backgrounds. Fig 7 shows the architecture of the CARAFE module.

First, the original input feature map is *I*(*H*,*W*,*C*), and the number of input channels *C* is compressed to using 1×1 convolution to *C*_*m*_ preserve the key information and reduce computational complexity. Next, a convolution operation is applied to the compressed feature map based on the information from the input feature map to generate the kernel for upsampling. Given a feature map χ of size C×H×W and an upsampling ratio of σ, nearest-neighbor interpolation produces a new feature map $\chi^{\prime }$ of size C×σH×σW. Each source position on χ corresponds to a target position $\sigma^{2}$ on $\chi^{\prime }$ and each target position requires an upsampling of $k_{up}\;\times \;k_{up}$ . This module generates a kernel of size $\sigma^{2}k_{up}^{2}\;\times \;H\;\times \;W$ for upsampling. The number of channels is expanded lalong the spatial dimension to obtain the kernel from upsampling kernel with a shape of $k_{up}^{2}\;\times \;\sigma H\;\times \;\sigma W$. Finally, the upsampling kernels are normalized.

The second step is the content reorganization module, which reorganizes the features using the generatedupsampling kernels. The reorganized feature map contains richer semantic information than the initial feature map. At any position $p^{\prime }=(i^{\prime },j^{\prime })$ of the output feature map, there is a corresponding source position p=(i,j) in the input feature map , where $i=\lfloor \frac{i^{\prime }}{\sigma }\rfloor$, $j=\lfloor \frac{j^{\prime }}{\sigma }\rfloor$ . In Fig 7, denotes the $k_{up}\;\times \;k_{up}$ subregion of centered at position *p* , and $W_{l}^{\prime }$denotes the upsampling kernel of *p* with size $k_{up}\times k_{up}$. Combining the dot product $N(\chi_{l},k_{up})$ with $W_{l}^{\prime }$ yields the final output value.

#### 3.2.3 SEAM module.

**Fig 8:**
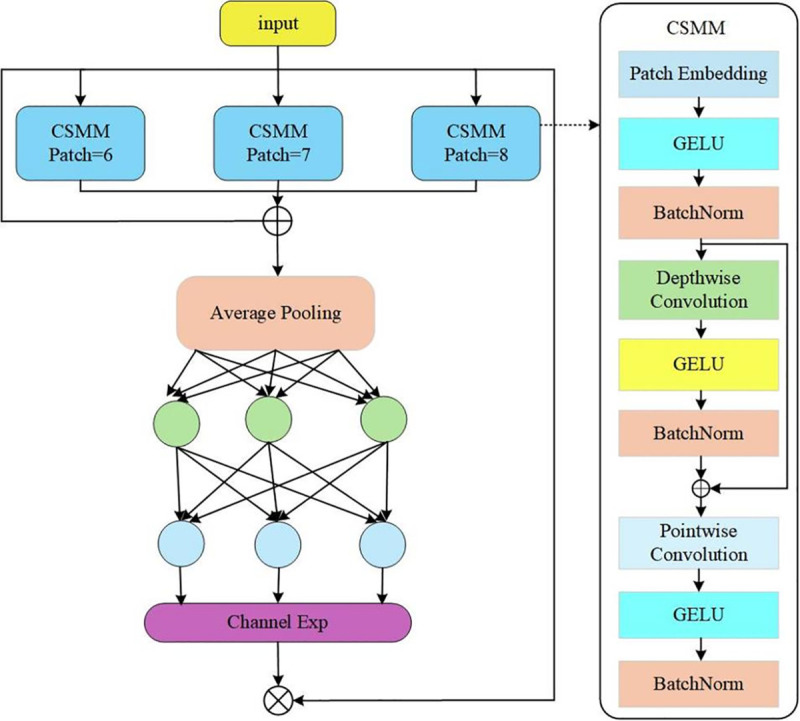
SEAM module diagram.

The ability of the model to accurately locate the license plate region may be reduced under complex interference conditions such as illumination variation and occlusion. To address this problem, this study introduces the SEAM [29] and applies it to the feature fusion stage of the YOLOv8 model. SEAM connects the three output layers at different scales in YOLOv8 with the three detection heads in the output section, thereby improving localization capability. Fig 8 shows the architecture of the SEAM.

First, SEAM employs depthwise separable convolution with a residual connection to learn the importance of different channel features while reducing the number of parameters. Next, the module integrates information across channels through a two-layer fully connected network, which enhances global channel relationships and enables the network to capture collaborative feature interactions more effectively. With SEAM, the model can identify important features more efficiently under conditions such as illumination change or occlusion, which improves localization accuracy and strengthens overall detection performance for license plates.

Fig 8 shows the block diagram of the SEAM module. SEAM consists of three Channel and Spatial Mixing Modules (CSMMs) of different sizes (Patch = 6, Patch = 7, and Patch = 8). The outputs from these modules are averaged, pooled, and processed through channel expansion, then multiplied to obtain enhanced feature representation. The CSMM module leverages multiscale features through patches of different sizes and uses depthwise separable convolution to learn spatial and channel correlations and embed the input patch. SEAM employs the GELU activation function to accelerate training and improve performance.

In the SEAM module, the exclusion loss function is a key component that enhances feature representation and detection accuracy under complex scenarios. Its primary role is to ensure that the model can effectively distinguish the license plate region from irrelevant background information during training. By encouraging the model to emphasize the license plate region while reducing dependence on background cues, the ELF helps limit background noise interference.

To address the issues of insufficient coverage and duplicate predictions in license plate detection, this paper introduces a composite loss function that integrates RepGT (Representation of Ground Truth Loss) with RepBox (Repulsion between Boxes Loss). The RepGT loss strengthens bounding box regression to ensure full coverage of license plate regions, while the RepBox loss regulates relationships among proposals during training to reduce redundant predictions of the same target. This dual-mechanism design improves the adaptability of the loss function for this task. The computational formulation of RepGT is shown in Eq (1), and the RepBox calculation is defined in Eq (2).

$${\text{Ł}}_{\text{RepGT}}=\frac{\sum_{P\in 𝒫}{\text{Smooth}}_{\text{ln}}(\text{IoU}(P,G_{\text{Rep}}^{P}))}{\left|P\right|}$$ (1)

$${\text{Ł}}_{\text{RepBox}}=\frac{\sum_{i\neq j}{\text{Smooth}}_{ln}(\text{IoU}(B_{pi},B_{pj}))}{\sum_{i\neq j}[\text{IoU}(B_{pi},B_{pj})>0]+\varepsilon }$$ (2)

The definition of $Smooth_{\ln}$ is as follows:

$$Smooth_{\ln}=\left\{ \begin{array}{cc} -\ln(1-x), & x\leq \sigma \\ \frac{x-\sigma }{1-\sigma }-\ln(1-\sigma ), & x>\sigma \end{array}\right.$$ (3)

In Eq (1), |P| represents the total number of candidate boxes and is used for normalization. The $Smooth_{\ln}$ function smooths the IoG (Intersection over Ground Truth) to increase the model’s sensitivity to gradient changes in low IoG regions while suppressing gradients in high IoG regions. $\sum_{p\in P}$ denotes the sum of all predicted boxes*P*, which is used to evaluate their matching quality with the ground truth boxes. $IoG(P,G_{Rep}^{p})$ represents the overlap (IoG) between the predicted box and the ground truth box. The IoG here is defined as $\frac{\text{area}(P\cap G)}{\text{area}(G)}$ , and its value ranges from 0 to 1, reflecting the proportion of the ground truth box that is covered by the predicted box.

In Eq (2), *B*_*pi*_ and *B*_*pj*_ represent prediction boxes *i* and *j*, respectively, where i≠j. The $IoU(B_{pi},B_{pj})$ is the calculated intersection over union (IoU) between the prediction boxes *B*_*pi*_ and *B*_*pj*_ , which measures the degree of overlap between the two bounding boxes. The value of IoU ranges from 0 to 1, with larger value indicating greater overlap. In complex license plate recognition scenarios, the RepBox loss function enhances detection quality by regulating the bounding box overlap. In addition, $1[IoU(B_{pi},B_{pj})>0]$ is an indicator function used to count the number of pairs of predicted boxes with an IoU greater than 0, and ε is a small constant used to prevent the denominator from becoming 0.

In Eq (3), σ serves as a smoothing parameter with a value range of (0, 1), regulating the sensitivity of the repulsion loss to outliers. Based on prior knowledge [29], ε is ultimately set to 0.1.

Compared with conventional IoU loss, RepGT uses the ground truth area as the denominator, making it more sensitive in cases where predicted bounding boxes do not fully cover the target or are smaller than the ground truth. For well-defined targets such as license plates, which require high localization accuracy, RepGT encourages predicted bounding boxes to provide complete coverage of the license plate region. Therefore, this study adopts a combined loss strategy that integrates RepGT with RepBox for license plate detection, addressing both localization completeness and prediction conciseness.

#### 3.2.4 C2fMLLABlock module.

MLLA [40] integrates two key elements of Mamba: the forget gate and module design. It replaces the forget gate with rotary position encoding (RoPE) to maintain parallel computation and inference speed while providing positional information.

The traditional forget gate mechanism supplies local positional cues but may reduce computational efficiency. To improve parallel computational performance, MLLABlock employs RoPE instead of forget gates. RoPE embeds relative positional information into features, allowing the model to preserve spatial information without compromising parallelism. This is particularly important for license plate detection because the position and orientation of a license plate vary within an image. Incorporating RoPE helps the model recognize license plates more accurately under different viewpoints and improves detection accuracy.

$$RoPE(x,\theta )=x\cdot \cos(\theta )+x_{⟂}\cdot \sin(\theta )$$ (4)

In Eq (4), the formula for calculating RoPE is presented, $x_{⟂}$ denotes the orthogonal component of the vector *x*, and θ is the encoded rotation angle, which is generated based on the input location *i*. Moreover, RoPE provides relative positional information and improves the model’s sensitivity to spatial structure.

**Fig 9:**
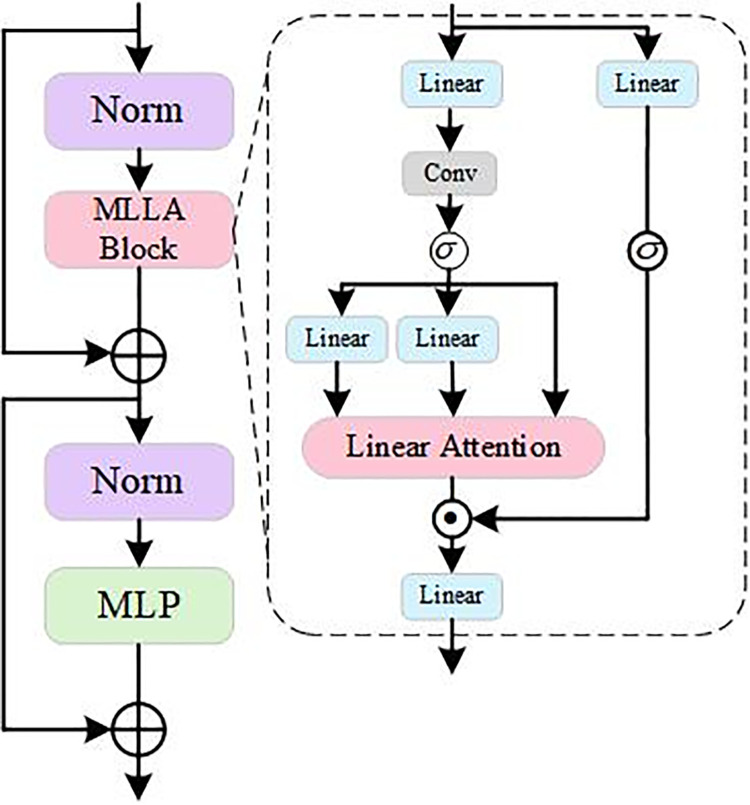
MLLABlock model architecture.

Through the multilevel attention mechanism of MLLABlock, C2fMLLABlock can dynamically adjust feature weights at different levels to extract key information across the feature pyramid. In the license plate recognition task, this multilevel attention mechanism highlights the feature representation of the license plate region, suppresses background interference and improves detection accuracy. The architectural structure of the MLLABlock is illustrated in Fig 9.

In complex scenarios, license plates are usually small targets that are strongly affected by environmental factors. Integrating C2fMLLABlock into YOLOv8 increases the model’s sensitivity to license plate detection. Through its multilevel attention mechanism, the model can weight license plate features at different scales. In scenarios with complex backgrounds or uneven lighting, the model focuses on the license plate region more accurately and reduces the false detection rate. The dynamic weight adjustment mechanism in MLLABlock further helps reduce the misrecognition of non-license plate regions.

### 3.3 CSM-LPRNet model

**Fig 10:**
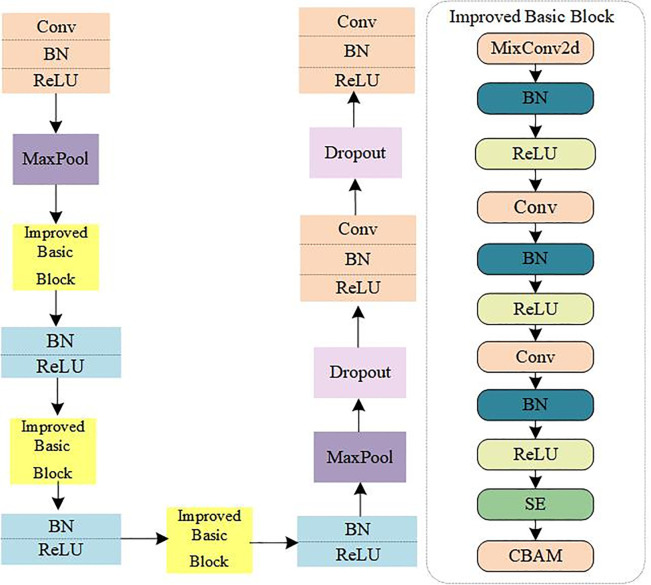
CSM-LPRNet network model.

**Fig 11:**
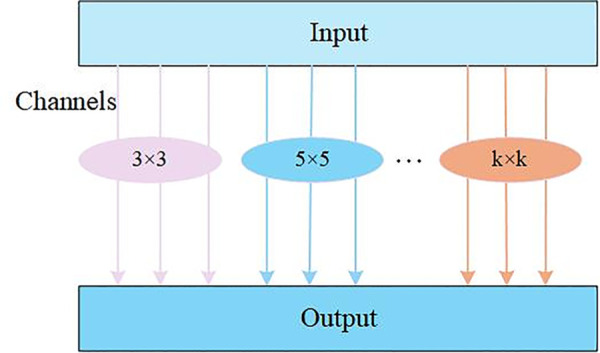
MixConv2d network model.

The LPRNet is a lightweight neural network architecture designed specifically for the task of license plate character recognition. It can quickly and accurately extract character information from detected license plate images [32]. The network structure of MixConv2d is shown Fig 11. Because its design eliminates the need for character segmentation, it greatly improves the processing speed and efficiency of character recognition. Fig 10 shows the architecture of the CSM-LPRNet network model. Its improved feature extraction module further enhances its ability to capture character features, maintaining high recognition accuracy and robustness in complex environments.

To extract this multiscale information more effectively, the MixConv2d module [40] was introduced. Traditional convolutional layers typically use a fixed-size convolutional kernel to extract features, whereas MixConv2d applies convolutional kernels of multiple sizes concurrently to perform convolution on the input features. This allows the module to capture multiscale information under different receptive fields. This design integrates local details with global contextual information and improves the feature representation ability of the model.

**Fig 12:**
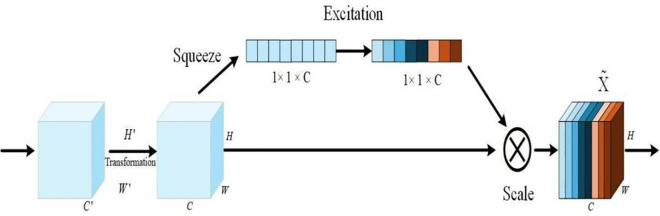
SE model architecturel.

In addition, features from different channels contribute unequally to the license plate recognition task. To utilize this channel information more effectively, the SE module is introduced to adaptively adjust channel weights [41]. The structure of the Squeeze-and-Excitation (SE) module is shown in Fig 12.

The adaptive feature allocation of SE is primarily achieved via Squeeze, Excitation, and Scale operations. It assigns weights based on how many target features are contained in the channels and simultaneously reduces the weights of non-target feature channels in the network to improve network performance. Fig 12. shows the SE-Net architecture.

The Squeeze operation computes the global mean of the input features along the spatial dimensions, as shown in (5):

$$z_{c}=\frac{1}{H\times W}\sum_{i=1}^{H}\sum_{j=1}^{W}X_{c,i,j}$$ (5)

Then *z* is passed through the Excitation operation, denoted by *F*_*ex*_ to obtain the feature weights of different feature channels *s*, as shown in 6:

$$S=F_{ex}(z,W)=\sigma (W_{2},\delta (W_{1},z))\;$$ (6)

where *W*_1_ and *W*_2_ denote the dimension of the fully connected layer, and σ and δ denote the Sigmoid and LeakyRelu functions, respectively.

The weights from the Excitation output can be regarded as the weights of each feature channel after feature selection. These feature weights are multiplied with their corresponding previous feature channels to complete the feature selection stage. After the scale operation, the weights of *S* denoted as *F*_*scale*_ are applied to *U*, and the output is $\tilde{X}$, as shown in 7:

$$\tilde{X}=F_{scale}(u_{c},s_{c})=s_{c}\cdot u_{c}$$ (7)

**Fig 13:**
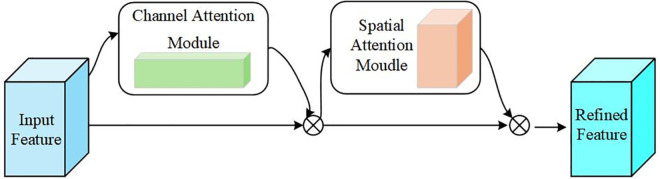
CBAM model architecture.

The structure of the CBAM module is shown in Fig 13. The CBAM module [30] combines channel attention with spatial attention to enhance the network’s ability to capture important information related to license plate characters.

This module first applies channel attention to weight each channel, followed by spatial attention to weight spatial locations in the image. The channel attention mechanism generates the feature vectors $Z_{avg}$ and *Z*_*max*_ hrough global average pooling and global maximum pooling, respectively.

$$Z_{\text{avg}}=\frac{1}{H\times W}\sum_{i=1}^{H}\sum_{j=1}^{W}X_{c,i,j}$$ (8)

Channel attention generates feature vectors through global average pooling and global maximum pooling, as shown in 9:

$$Z_{\max}=\max_{i,j}X_{c,i,j}\;$$ (9)

Next, these vectors are concatenated along the channel dimension and passed through a 7×7 convolution to generate the spatial attention weights *S*_*s*_, as shown in 10:

$$S_{s}=\sigma (\text{Conv}7\times 7([X_{avg}^{\prime },X_{max}^{\prime }]))\;$$ (10)

Finally, these spatial weights are applied to the feature map through element-by-element multiplication to obtain the weighted output $X^{\prime \prime }$:

$$X^{\prime \prime }=S_{s}\cdot X^{\prime }$$ (11)

In this study, the Improved Basic Block is designed by integrating MixConv2d, an SE module and a CBAM module. Given the input feature map *X*, the Improved Basic Block first extracts multiscale features through MixConv2d, then applies channel and spatial weighting through the SE and CBAM modules, respectively. Finally, the output is added to the original input through residual linking:

Y=X+CBAM(SE(MixConv2d(X))) (12)

To further improve the accuracy of license plate recognition, this study introduces global contextual information integration. At key layers of the network, the feature maps are retained, and global average pooling is used to compress the high-dimensional feature maps to a fixed size. Assuming that each feature map is *f*_*i*_, square and average pooling operations are applied to generate the global context features *g*_*i*_, as shown in 13:

$$g_{i}=\frac{f_{i}}{\text{mean}(f_{i}^{2})}$$ (13)

These context features capture global information and are connected at subsequent layers to form the final global feature *g*, as shown in 14:

$$g=\text{Concat}(g_{1},g_{2},\ldots ,g_{n})$$ (14)

## 4 Analysis of results

In training the CSM-LPRNet network in this study, the AdamW optimizer [42] is used, the learning rate is set to 0.01 and the maximum training period is set as . The learning rate decays according to a cosine curve during each training period, as given in 15:

$$\eta_{t}=\eta_{\min}+\frac{1}{2}(\eta_{\max}-\eta_{\min})\left(1+\cos\left(\frac{T_{\text{cur}}}{T_{\text{max}}}\pi \right)\right)$$ (15)

where $\eta_{t}$ is the current learning rate; $\eta_{\min}$ and $\eta_{\max}$ are the minimum and maximum values of the learning rate; respectively; $T_{\text{cur}}$is the current training step; and $T_{\text{max}}$ is the maximum number of steps in the learning rate cycle.

In this study, the AdamW optimizer is used in conjunction with the cosine annealing learning rate scheduler [43] to allow the model to converge quickly during training while reducing the risk of falling into local optima as the learning rate decreases in the later stages. In addition, the regularization effect of AdamW suppresses overfitting and helps the model achieve better generalization ability. Moreover, the experiment used 2,368 images, and detailed annotations were provided for each sample to ensure dataset adequacy and reproducibility of the results.

### 4.1 Experimental setup

In this study, 2,368 images were selected based on the image classifications from the CCPD2019 dataset [44], including CCPD_weather,CCPD_blur and CCPD_db. The images were randomly allocated to the training, validation, and test sets at a ratio of 7:2:1. Table 1 shows the distribution of the different environmental datasets. The random seed was fixed at 11 throughout the experiments to ensure reproducible results. To improve the model’s adaptability to complex environments, a set of data augmentation strategies was applied during training, including hue adjustment, brightness transformation, random rotation, and scaling. In addition, a 5-fold cross-validation approach was used to evaluate model performance, improving the stability and reliability of the assessment results.

**Table 1:** Experimental environment configuration.

Scenario type	Snowy day	Rainy day	Foggy day	Dim light	Bright light
Count	480	480	549	384	475

### 4.2 Experimental evaluation metrics

Precision (P), Recall (R), Accuracy (AP), and Mean Accuracy (mAP) are used as performance evaluation metrics [45]. The specific formulas are given in 16–19:

$$P=\frac{TP}{TP+FP}$$ (16)

$$R=\frac{TP}{TP+FN}$$ (17)

$$AP=\frac{TP}{N(TP+FP)}$$ (18)

$$mAP=\frac{1}{n}\sum_{k=1}^{n}AP_{k}$$ (19)

Here, TP is the number of correctly predicted positive samples, FP is the number of negative samples predicted as positive samples, FN is the number of positive samples predicted as negative samples, and N is the total number of samples.

### 4.3 Experimental environment

The experimental platform used in this study was built on the Windows operating system. The experiments were conducted using Python 3.11, based on the PyTorch 1.13.1 framework, and accelerated by CUDA 11.6 and cuDNN 8.2 for model training and inference, as shown in Table 2 .

**Table 2:** Experimental configuration.

Configuration of the experiment	Specification
Operating system	Windows 11
CPU	AMD EPYC 7601 32-Core Processor
GPU	NVIDIA RTX4070
IDE	PyCharm
Deep learning framework	PyTorch 1.13.1
CUDA version	CUDA11.7

### 4.4 Experimental environment

During the training of the CSCM-YOLOv8 model, specific hyperparameters were defined to optimize performance, as shown in Table 3 .

**Table 3:** Experimental configuration.

Training parameter	Value
Epochs	100
Batch	16
Initial learning rate	0.001
Momentum	0.937
Weight_decay	0.5
Optimizer	SGD

## 5 Experimental results and analysis

### 5.1 License plate detection experiment

**Fig 14:**
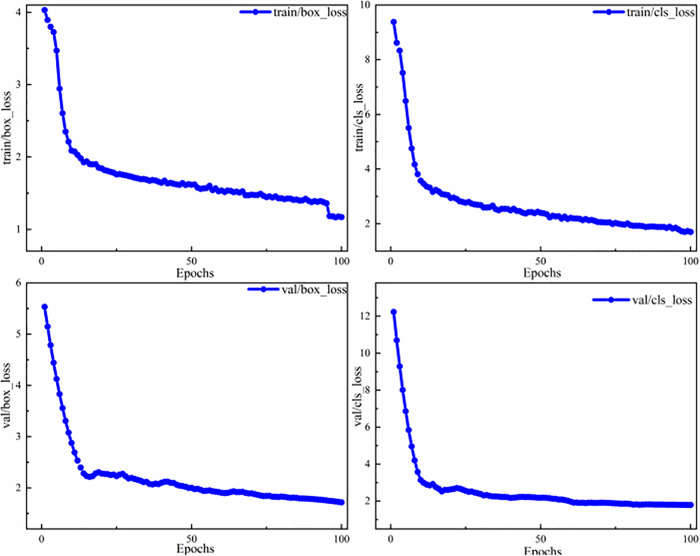
Loss function of the CSCM-YOLOv8 network on the license plate dataset.

Fig 14 shows the changes in the loss function of the proposed network on the license plate dataset, including performance metrics for both the training and validation sets as well as two different classes of loss functions. Here, train/box_loss is the bounding box regression loss computed during the training phase and measures the error between the predicted and ground truth bounding boxes. A decrease in this loss indicates that the model gradually improves its target localization capability. The train/cls_loss is the classification loss during the training phase and measures the difference between the class probability distribution predicted by the model and the ground truth labels. The val/box_loss is the bounding box regression loss calculated during the validation phase and reflects the error between the predicted and ground truth boxes on the validation set. The val/cls_loss is the classification loss during the validation phase and represents the difference between the predicted class probabilities and the true labels on the validation set [46].

**Fig 15:**
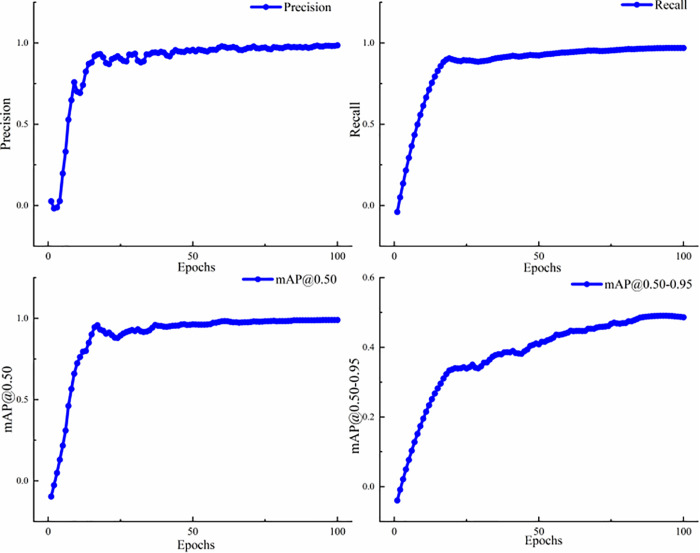
Evaluation metrics of the CSCM-YOLOv8 network on the license plate dataset.

Fig 15 illustrates the evaluation metrics of CSCM-YOLOv8 on the license plate dataset. The mAP@0.5 (mean average precision at IoU 0.5) denotes the mean value of the average precision of the model at an IoU threshold of 50%. A higher AP@0.5 indicates that the model performs well in detecting objects because it can accurately locate most of the targets when a 50% overlap between the detection and ground truth boxes is required. Moreover, mAP@0.5–0.95 represents the mean average precision (mAP) averaged over the IoU threshold range of 50% to 95%. The detection quality of the model is measured by calculating average precision at different IoU thresholds and then averaging these precision values [45]. A higher mAP@0.5–0.95 indicates that the model can accurately detect objects across different IoU thresholds and represents better generalization ability and precision.

As shown by the change curves of the four evaluation indexes in Fig 15, the detection accuracy increases steadily and eventually stabilizes at 98.9%, indicating that the improved network not only converges quickly during training but also maintains high performance in terms of learning stability.

**Fig 16:**
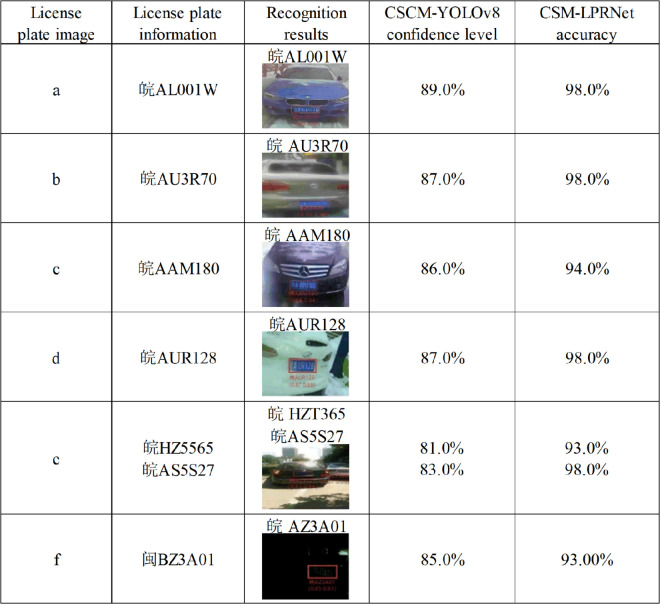
License plate recognition performance analysis of CSCM-YOLOv8 and CSM-LPRNet.

Fig 16 shows the recognition results of license plates in complex environments described in the problem analysis. These results demonstrate that the method proposed in this study achieves significant improvements in both detection and recognition performance in complex environments. The CSCM-YOLOv8 model shows higher detection confidence in localizing the license plate region, and CSM-LPRNet maintains a high recognition rate, further demonstrating that its character feature extraction accuracy is substantially enhanced.

### 5.2 Comparison of the performance of different detection methods

To compare the performance of different target detection algorithms, the experimental results of several mainstream detection methods are listed in Table 4, using the commonly used evaluation metrics of Precision, Recall, and Average Precision. Through these comparisons, the purpose is to validate the performance advantages of the method proposed in this study (Ours) in the task of license plate detection.

**Table 4:** Experimental results of a performance comparison of different target detection methods.

Method	Precision	Recall	mAP@0.50	mAP@0.50–0.95
YOLOv8 [2]	95.8%	97.1%	98.0%	54.1%
YOLOv7 [17]	94.2%	95.2%	96.0%	45.2%
YOLOv5 [47]	90.5%	91.9%	96.2%	47.3%
RT-DETR [45]	89.1%	90.1%	96.0%	49.3%
Faster-RCNN [48]	90.3%	90.3%	96.2%	48.2%
Ours	98.9%	98.0%	99.9%	58.0%

The performance comparison results presented in Table 4 demonstrate that the proposed method offers clear advantages across key evaluation metrics. In terms of accuracy, our method reaches 98.9%, a 3.1% improvement over YOLOv8, demonstrating stronger performance in reducing false positives. For recall, the method attains 98.0%, outperforming other approaches by 0.9% to 7.9%, which highlights its effectiveness in minimizing false negatives. Regarding comprehensive metrics, the proposed method achieves the highest mAP@0.50 value of 99.9%, exceeding the comparison methods by 1.9% to 3.9%. For mAP@0.50-0.95, our method achieves 58.0%, surpassing all comparison methods and improving upon YOLOv8 by 3.9%. These results indicate that the proposed method maintains stable performance across varying IoU thresholds while offering notable advantages in scenarios that require high localization accuracy.

**Fig 17:**
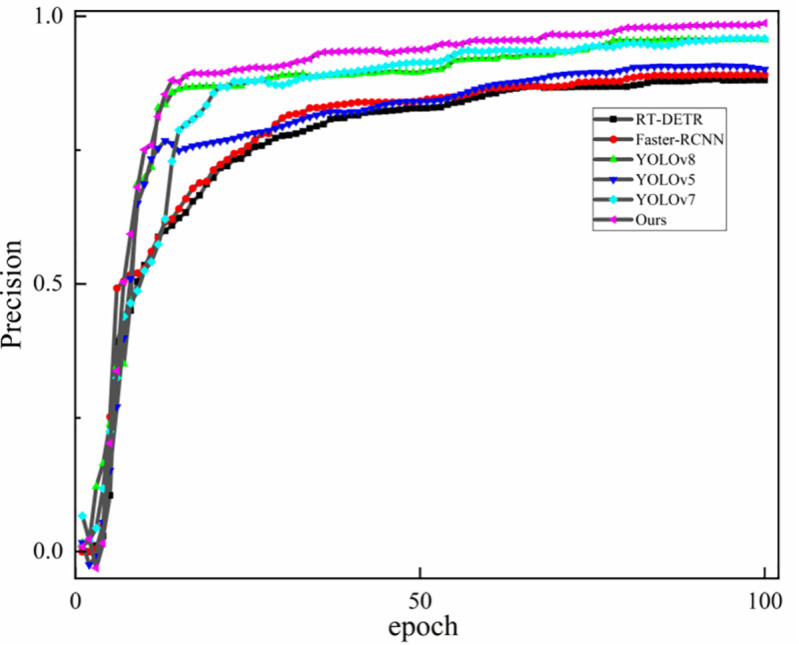
Accuracy convergence comparison of different models during training.

To objectively evaluate the performance advantages of the proposed method, Fig 17 shows the accuracy convergence curves during training for YOLOv8 [2], YOLOv7 [17], YOLOv5 [47], RT-DETR [45], Faster-RCNN [48], and the proposed method.

As shown in Fig 17, the training curves indicate that the accuracy of the proposed method increases steadily with each epoch. After 30 epochs, it exceeds all comparison models, including RT-DETR, Faster-RCNN and the YOLO series, and continues to outperform the competing methods in later training stages. These results suggest that the proposed method reduces the false positive rate and provides strong detection reliability in complex scenarios.

### 5.3 License plate detection ablation experiments

To further validate the improvements of CSCM-YOLOv8, a series of ablation experiments was conducted. By integrating different combinations of modules, including CPA-Enhancer, CARAFE, SEAM and MLLABlock, into YOLOv8, vehicle license plate detection experiments were carried out on the same dataset. The results are presented in Table 5.

**Table 5:** Ablation results of license plate detection experiments.

Method	Precision	Recall	mAP@0.50	mAP@0.50–0.95
Yolov8-MLLBlock	94.0%	97.3%	97.3%	54.1%
CPA-YOLOv8	96.2%	97.6%	98.6%	61.3%
Yolov8-SEAM	95.5%	97.6%	97.6%	57.3%
YOLOv8-CARAFE	96.4%	98.4%	97.9%	55.2%
CPA-Yolov8-CARAFE-SEAM	95.5%	93.6%	96.6%	51.3%
CPA-YOLOv8-CARAFE	97.0%	97.7%	98.6%	63.5%
CPA-YOLOv8-SEAM	97.6%	98.7%	98.6%	64.9%
CPA-YOLOv8-MLLABlock	97.9%	97.3%	98.3%	55.0%
CPA-YOLOv8-CARAFE-MLLABlock	98.0%	97.2%	97.8%	56.2%
YOLOv8-SEAM-MLLABlock	97.0%	97.8%	97.7%	53.3%
YOLOv8-CARAFE-SEAM	96.7%	95.8%	98.6%	55.3%
YOLOv8-CARAFE-MLLABlcok	96.6%	96.8%	96.2%	53.9%
YOLOv8-CARAFE-SEAM-MLLABlock	97.6%	96.8%	96.6%	57.8%
Ours	98.9%	98.0%	99.9%	58.0%

As shown in Table 5, the proposed method achieves the highest values among all models, reaching 99.9% in mAP@0.50 and 98.9% in accuracy, which indicates strong control of false positives. For mAP@0.50:0.95, the CPA-YOLOv8-SEAM model achieves64.9%, the best result across all models, while our method reaches 58.0%, surpassing most comparison methods and reflecting strong overall performance.

By examining the performance of different module combinations, the introduction of the CPA-Enhancer module has an evident impact on the mAP@0.50:0.95 metric. As shown in Table 5 shows, adding the CPA module to the YOLOv8-SEAM base increases mAP@0.50:0.95 from 57.3% to 64.9%. Similarly, adding the CPA module to the YOLOv8-CARAFE model raises the mAP@0.50:0.95 from 55.2% to 63.5%. These results suggest that the CPA-Enhancer module improves the model’s target localization accuracy. In addition, the inclusion of the SEAM module contributes to higher recall, with the CPA-YOLOv8-SEAM model achieving the highest recall value of 98.7% indicating that this module helps reduce false negatives. In contrast, simpler module combinations such as CPA-YOLOv8-SEAM-MLLABlock did not produce strong results, yielding an mAP@0.50:0.95 of only 53.3%. In comparison, the proposed method maintains the highest mAP@0.50 and accuracy values and also performs well in the mAP@0.50:0.95 metric. These findings support the effectiveness of the integrated strategy proposed in this paper, indicating that the architectural design enables the modules to work synergistically rather than functioning as a simple stack of components.

**Fig 18:**
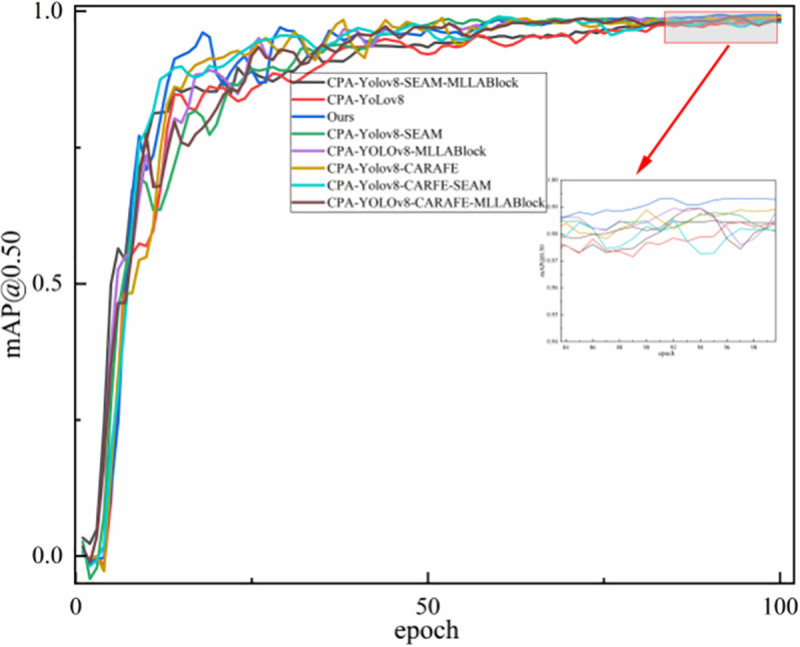
Convergence comparison of the mAP@0.50 values of different models during training.

To further evaluate performance in the license plate detection task, Fig 18 shows the mAP@0.50 curve across training epochs. By comparing the CPA-YOLOv8 baseline with its improved variants, the effectiveness and stability of the proposed method are further confirmed.

As shown in Fig 18, the curve trends indicate that the proposed method converges rapidly in the early training stages, with its performance metrics improving steadily. During the mid-training phase, the model maintains a stable upward trend without noticeable fluctuations. By the later stages of training, the model has essentially converged, and its final performance surpasses all comparison models. These results suggest that the proposed method achieves efficient training and, through effective module fusion, delivers stronger final performance than the other models.

**Fig 19:**
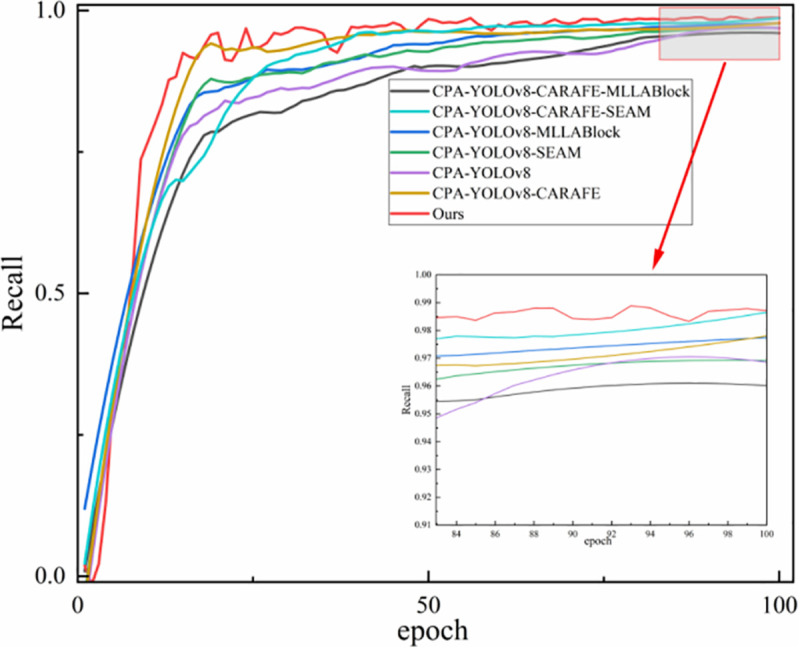
Convergence curves of the recall rates of different models during the training process.

Fig 19 shows the recall curves across training epochs for different models, which helps assess how each method’s target-capturing ability evolves throughout the training process.

From the training curve shown in Fig 19, the recall rate of the proposed method improves steadily with each epoch and reaches convergence in the later training stages. The final recall value exceeds that of all comparison models. These findings suggest that the proposed method is more effective at detecting true targets and substantially reduces the false negative rate.

### 5.4 Comparison of license plate recognition experiments

By comparing the performance of the LPRNet baseline model and the simple combinations of modules with the method proposed in this study, the independent contribution of each module and their synergistic effects can be analyzed, allowing the scientific rigor and effectiveness of the method design in this study to be validated.

As shown in the results of Table 6, the network with the SE module achieves a 0.32% increase in accuracy compared with the original LPRNet, indicating that the SE module is more effective in highlighting the features of the character region and reducing the interference from irrelevant regions in complex scenarios. After introducing the CBAM module, the network accuracy reaches 90.34%, further improving upon the SE module alone and indicating that the CBAM enhances the model’s ability to distinguish characters through fine-grained processing of the character region. The network with the MixConv2d module achieves an accuracy of 90.49% allowing the model to handle diverse character patterns more comprehensively. The network proposed in this study uses a combination of SE, CBAM, and MixConv2d to leverage the strengths of each module across different feature dimensions. The accuracy reaches 98.56%, representing a 9.08% improvement over the original LPRNet and fully validating the effectiveness of these modules in character recognition.

**Table 6:** Comparison of license plate recognition experiments.

Method	Accuracy
LPRNet	89.48%
SE=F, CBAM=T, MixConv2d=F	90.34%
CBAM=F, SE=T, MixConv2d=F	89.80%
MixConv2d=T, CBAM=F, SE=F	90.49%
Ours	98.56%

### 5.5 Comparison of upsampling methods

To evaluate the practical performance of CARAFE in the license plate detection task, systematic comparison experiments were conducted. Using the same dataset and training settings, CARAFE was compared with the default Nearest [49] upsampling method used in YOLOv8 and with typical upsampling methods such as Bilinear [50] and Transpose Conv [51]. The results are shown in Table 7.

**Table 7:** Performance comparison of different upsampling methods on the license plate detection dataset.

Upsampling Methods	Upsampling Methods	mAP@0.50	Recall
Nearest [49]	95.8%	98.0%	97.1%
Bilinear [50]	94.2%	96.0%	95.8%
Transpose Conv [51]	93.0%	95.2%	97.1%
CARAFE	96.4%	97.9%	98.4%

The experimental results in Table 7 showthat the CARAFE method used in this paper reaches 96.4% in accuracy, outperforming Nearest Neighbor interpolation, Bilinear interpolation and Transpose Convolution by 0.6%, 2.2%, and 3.4%, respectively. This indicates a clear advantage in reducing false positives. For mAP@0.50, our method achieves 97.9%, exceeding the other comparison methods. For recall, the method reaches 98.4%, the highest among all models.

From an overall performance perspective, the CARAFE method outperforms the comparison models in Precision, mAP@0.50, and Recall, suggesting that it balances spatial details and semantic information more effectively in feature maps and improves feature representation quality. In contrast, traditional interpolation methods show weaker performance in accuracy and mAP. Although Transpose Convolution performs similarly to Nearest Neighbor interpolation in recall, it falls notably behind in Precision and mAP.

## 6 Conclusion

The CSCM-YOLOv8 and CSM-LPRNet networks proposed in this study achieve significant performance improvements in license plate detection and recognition tasks. To address the low accuracy of license plate detection under rainy, snowy, and complex lighting conditions, YOLOv8 enhances detection robustness by incorporating the CPA-Enhancer, SEAM, CARAFE, and C2fMLLABlock modules. For license plate character recognition, LPRNet improves the model’s ability to recognize characters in complex scenarios by integrating the SE, CBAM and MixConv2d modules. The experimental results show that, compared with the original network, the model proposed in this study achieves improvements of 3.1% in accuracy and 3.9% in mAP@0.50–0.95. These improvements validate the effectiveness of the proposed method for handling license plate detection and recognition tasks in complex environments. Future research will continue to explore further optimization of this network, including porting the model to an edge mobile platform for validation and refinement to enhance lightweight characteristics and improve deployment efficiency in practical applications.

## Supporting information

S1 Data (ZIP)
