# Supplementary figures and images for: License plate recognition methodology in complex scenarios based on CSCM-YOLOv8 and CSM-LPRNet (part 2 of 2)
Source: PLoS One. 2026 Jan 2;21(1):e0339649. doi: 10.1371/journal.pone.0339649 (PMC12758793; doi:10.1371/journal.pone.0339649)

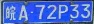

Supplement: S1 Data — (ZIP) [file pone.0339649.s001.zip › S1 Data/train/皖A72P33.jpg]

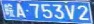

Supplement: S1 Data — (ZIP) [file pone.0339649.s001.zip › S1 Data/train/皖A753V2.jpg]

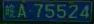

Supplement: S1 Data — (ZIP) [file pone.0339649.s001.zip › S1 Data/train/皖A75524.jpg]

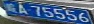

Supplement: S1 Data — (ZIP) [file pone.0339649.s001.zip › S1 Data/train/皖A75556.jpg]

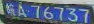

Supplement: S1 Data — (ZIP) [file pone.0339649.s001.zip › S1 Data/train/皖A76737.jpg]

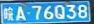

Supplement: S1 Data — (ZIP) [file pone.0339649.s001.zip › S1 Data/train/皖A76Q38.jpg]

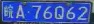

Supplement: S1 Data — (ZIP) [file pone.0339649.s001.zip › S1 Data/train/皖A76Q62.jpg]

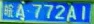

Supplement: S1 Data — (ZIP) [file pone.0339649.s001.zip › S1 Data/train/皖A772A1.jpg]

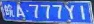

Supplement: S1 Data — (ZIP) [file pone.0339649.s001.zip › S1 Data/train/皖A777Y1.jpg]

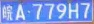

Supplement: S1 Data — (ZIP) [file pone.0339649.s001.zip › S1 Data/train/皖A779H7.jpg]

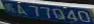

Supplement: S1 Data — (ZIP) [file pone.0339649.s001.zip › S1 Data/train/皖A77Q40.jpg]

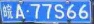

Supplement: S1 Data — (ZIP) [file pone.0339649.s001.zip › S1 Data/train/皖A77S66.jpg]

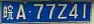

Supplement: S1 Data — (ZIP) [file pone.0339649.s001.zip › S1 Data/train/皖A77Z41.jpg]

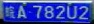

Supplement: S1 Data — (ZIP) [file pone.0339649.s001.zip › S1 Data/train/皖A782U2.jpg]

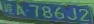

Supplement: S1 Data — (ZIP) [file pone.0339649.s001.zip › S1 Data/train/皖A786J2.jpg]

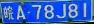

Supplement: S1 Data — (ZIP) [file pone.0339649.s001.zip › S1 Data/train/皖A78J81.jpg]

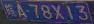

Supplement: S1 Data — (ZIP) [file pone.0339649.s001.zip › S1 Data/train/皖A78X13.jpg]

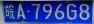

Supplement: S1 Data — (ZIP) [file pone.0339649.s001.zip › S1 Data/train/皖A796G8.jpg]

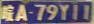

Supplement: S1 Data — (ZIP) [file pone.0339649.s001.zip › S1 Data/train/皖A79Y11.jpg]

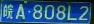

Supplement: S1 Data — (ZIP) [file pone.0339649.s001.zip › S1 Data/train/皖A808L2.jpg]

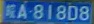

Supplement: S1 Data — (ZIP) [file pone.0339649.s001.zip › S1 Data/train/皖A818D8.jpg]

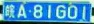

Supplement: S1 Data — (ZIP) [file pone.0339649.s001.zip › S1 Data/train/皖A81G01.jpg]

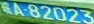

Supplement: S1 Data — (ZIP) [file pone.0339649.s001.zip › S1 Data/train/皖A82023.jpg]

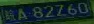

Supplement: S1 Data — (ZIP) [file pone.0339649.s001.zip › S1 Data/train/皖A82Z60.jpg]

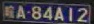

Supplement: S1 Data — (ZIP) [file pone.0339649.s001.zip › S1 Data/train/皖A84A12.jpg]

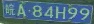

Supplement: S1 Data — (ZIP) [file pone.0339649.s001.zip › S1 Data/train/皖A84H99.jpg]

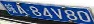

Supplement: S1 Data — (ZIP) [file pone.0339649.s001.zip › S1 Data/train/皖A84V80.jpg]

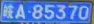

Supplement: S1 Data — (ZIP) [file pone.0339649.s001.zip › S1 Data/train/皖A85370.jpg]

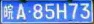

Supplement: S1 Data — (ZIP) [file pone.0339649.s001.zip › S1 Data/train/皖A85H73.jpg]

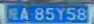

Supplement: S1 Data — (ZIP) [file pone.0339649.s001.zip › S1 Data/train/皖A85Y58.jpg]

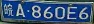

Supplement: S1 Data — (ZIP) [file pone.0339649.s001.zip › S1 Data/train/皖A860E6.jpg]

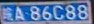

Supplement: S1 Data — (ZIP) [file pone.0339649.s001.zip › S1 Data/train/皖A86C88.jpg]

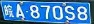

Supplement: S1 Data — (ZIP) [file pone.0339649.s001.zip › S1 Data/train/皖A870S8.jpg]

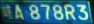

Supplement: S1 Data — (ZIP) [file pone.0339649.s001.zip › S1 Data/train/皖A878R3.jpg]

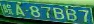

Supplement: S1 Data — (ZIP) [file pone.0339649.s001.zip › S1 Data/train/皖A87BB7.jpg]

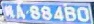

Supplement: S1 Data — (ZIP) [file pone.0339649.s001.zip › S1 Data/train/皖A884B0.jpg]

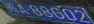

Supplement: S1 Data — (ZIP) [file pone.0339649.s001.zip › S1 Data/train/皖A88G02.jpg]

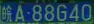

Supplement: S1 Data — (ZIP) [file pone.0339649.s001.zip › S1 Data/train/皖A88G40.jpg]

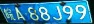

Supplement: S1 Data — (ZIP) [file pone.0339649.s001.zip › S1 Data/train/皖A88J99.jpg]

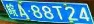

Supplement: S1 Data — (ZIP) [file pone.0339649.s001.zip › S1 Data/train/皖A88T24.jpg]

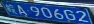

Supplement: S1 Data — (ZIP) [file pone.0339649.s001.zip › S1 Data/train/皖A906G2.jpg]

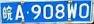

Supplement: S1 Data — (ZIP) [file pone.0339649.s001.zip › S1 Data/train/皖A908W0.jpg]

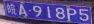

Supplement: S1 Data — (ZIP) [file pone.0339649.s001.zip › S1 Data/train/皖A918P5.jpg]

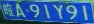

Supplement: S1 Data — (ZIP) [file pone.0339649.s001.zip › S1 Data/train/皖A91Y91.jpg]

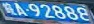

Supplement: S1 Data — (ZIP) [file pone.0339649.s001.zip › S1 Data/train/皖A92888.jpg]

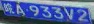

Supplement: S1 Data — (ZIP) [file pone.0339649.s001.zip › S1 Data/train/皖A933V2.jpg]

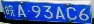

Supplement: S1 Data — (ZIP) [file pone.0339649.s001.zip › S1 Data/train/皖A93AC6.jpg]

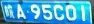

Supplement: S1 Data — (ZIP) [file pone.0339649.s001.zip › S1 Data/train/皖A95C01.jpg]

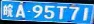

Supplement: S1 Data — (ZIP) [file pone.0339649.s001.zip › S1 Data/train/皖A95T71.jpg]

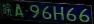

Supplement: S1 Data — (ZIP) [file pone.0339649.s001.zip › S1 Data/train/皖A96H66.jpg]

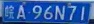

Supplement: S1 Data — (ZIP) [file pone.0339649.s001.zip › S1 Data/train/皖A96N71.jpg]

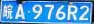

Supplement: S1 Data — (ZIP) [file pone.0339649.s001.zip › S1 Data/train/皖A976R2.jpg]

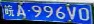

Supplement: S1 Data — (ZIP) [file pone.0339649.s001.zip › S1 Data/train/皖A996V0.jpg]

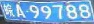

Supplement: S1 Data — (ZIP) [file pone.0339649.s001.zip › S1 Data/train/皖A99788.jpg]

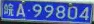

Supplement: S1 Data — (ZIP) [file pone.0339649.s001.zip › S1 Data/train/皖A99804.jpg]

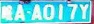

Supplement: S1 Data — (ZIP) [file pone.0339649.s001.zip › S1 Data/train/皖AA017Y.jpg]

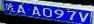

Supplement: S1 Data — (ZIP) [file pone.0339649.s001.zip › S1 Data/train/皖AA097V.jpg]

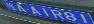

Supplement: S1 Data — (ZIP) [file pone.0339649.s001.zip › S1 Data/train/皖AA1R81.jpg]

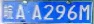

Supplement: S1 Data — (ZIP) [file pone.0339649.s001.zip › S1 Data/train/皖AA296M.jpg]

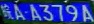

Supplement: S1 Data — (ZIP) [file pone.0339649.s001.zip › S1 Data/train/皖AA379A.jpg]

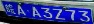

Supplement: S1 Data — (ZIP) [file pone.0339649.s001.zip › S1 Data/train/皖AA3Z73.jpg]

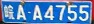

Supplement: S1 Data — (ZIP) [file pone.0339649.s001.zip › S1 Data/train/皖AA4755.jpg]

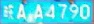

Supplement: S1 Data — (ZIP) [file pone.0339649.s001.zip › S1 Data/train/皖AA4790.jpg]

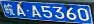

Supplement: S1 Data — (ZIP) [file pone.0339649.s001.zip › S1 Data/train/皖AA5360.jpg]

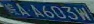

Supplement: S1 Data — (ZIP) [file pone.0339649.s001.zip › S1 Data/train/皖AA603W.jpg]

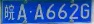

Supplement: S1 Data — (ZIP) [file pone.0339649.s001.zip › S1 Data/train/皖AA662G.jpg]

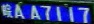

Supplement: S1 Data — (ZIP) [file pone.0339649.s001.zip › S1 Data/train/皖AA7117.jpg]

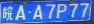

Supplement: S1 Data — (ZIP) [file pone.0339649.s001.zip › S1 Data/train/皖AA7P77.jpg]

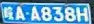

Supplement: S1 Data — (ZIP) [file pone.0339649.s001.zip › S1 Data/train/皖AA838H.jpg]

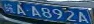

Supplement: S1 Data — (ZIP) [file pone.0339649.s001.zip › S1 Data/train/皖AA892A.jpg]

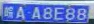

Supplement: S1 Data — (ZIP) [file pone.0339649.s001.zip › S1 Data/train/皖AA8E88.jpg]

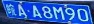

Supplement: S1 Data — (ZIP) [file pone.0339649.s001.zip › S1 Data/train/皖AA8M90.jpg]

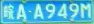

Supplement: S1 Data — (ZIP) [file pone.0339649.s001.zip › S1 Data/train/皖AA949M.jpg]

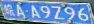

Supplement: S1 Data — (ZIP) [file pone.0339649.s001.zip › S1 Data/train/皖AA9Z96.jpg]

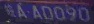

Supplement: S1 Data — (ZIP) [file pone.0339649.s001.zip › S1 Data/train/皖AAD090.jpg]

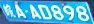

Supplement: S1 Data — (ZIP) [file pone.0339649.s001.zip › S1 Data/train/皖AAD898.jpg]

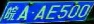

Supplement: S1 Data — (ZIP) [file pone.0339649.s001.zip › S1 Data/train/皖AAE500.jpg]

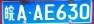

Supplement: S1 Data — (ZIP) [file pone.0339649.s001.zip › S1 Data/train/皖AAE630.jpg]

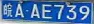

Supplement: S1 Data — (ZIP) [file pone.0339649.s001.zip › S1 Data/train/皖AAE739.jpg]

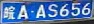

Supplement: S1 Data — (ZIP) [file pone.0339649.s001.zip › S1 Data/train/皖AAS656.jpg]

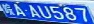

Supplement: S1 Data — (ZIP) [file pone.0339649.s001.zip › S1 Data/train/皖AAU587.jpg]

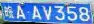

Supplement: S1 Data — (ZIP) [file pone.0339649.s001.zip › S1 Data/train/皖AAV358.jpg]

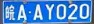

Supplement: S1 Data — (ZIP) [file pone.0339649.s001.zip › S1 Data/train/皖AAY020.jpg]

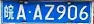

Supplement: S1 Data — (ZIP) [file pone.0339649.s001.zip › S1 Data/train/皖AAZ906.jpg]

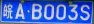

Supplement: S1 Data — (ZIP) [file pone.0339649.s001.zip › S1 Data/train/皖AB003S.jpg]

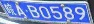

Supplement: S1 Data — (ZIP) [file pone.0339649.s001.zip › S1 Data/train/皖AB0589.jpg]

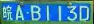

Supplement: S1 Data — (ZIP) [file pone.0339649.s001.zip › S1 Data/train/皖AB113D.jpg]

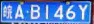

Supplement: S1 Data — (ZIP) [file pone.0339649.s001.zip › S1 Data/train/皖AB146Y.jpg]

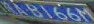

Supplement: S1 Data — (ZIP) [file pone.0339649.s001.zip › S1 Data/train/皖AB166M.jpg]
