# Supplementary figures and images for: License plate recognition methodology in complex scenarios based on CSCM-YOLOv8 and CSM-LPRNet (part 1 of 2)
Source: PLoS One. 2026 Jan 2;21(1):e0339649. doi: 10.1371/journal.pone.0339649 (PMC12758793; doi:10.1371/journal.pone.0339649)

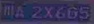

Supplement: S1 Data — (ZIP) [file pone.0339649.s001.zip › S1 Data/kkkk.jpg]

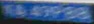

Supplement: S1 Data — (ZIP) [file pone.0339649.s001.zip › S1 Data/liang.jpg]

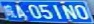

Supplement: S1 Data — (ZIP) [file pone.0339649.s001.zip › S1 Data/train/皖A051N0.jpg]

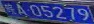

Supplement: S1 Data — (ZIP) [file pone.0339649.s001.zip › S1 Data/train/皖A05279.jpg]

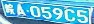

Supplement: S1 Data — (ZIP) [file pone.0339649.s001.zip › S1 Data/train/皖A059C5.jpg]

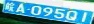

Supplement: S1 Data — (ZIP) [file pone.0339649.s001.zip › S1 Data/train/皖A095Q1.jpg]

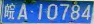

Supplement: S1 Data — (ZIP) [file pone.0339649.s001.zip › S1 Data/train/皖A10784.jpg]

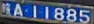

Supplement: S1 Data — (ZIP) [file pone.0339649.s001.zip › S1 Data/train/皖A11885.jpg]

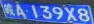

Supplement: S1 Data — (ZIP) [file pone.0339649.s001.zip › S1 Data/train/皖A139X8.jpg]

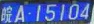

Supplement: S1 Data — (ZIP) [file pone.0339649.s001.zip › S1 Data/train/皖A15104.jpg]

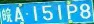

Supplement: S1 Data — (ZIP) [file pone.0339649.s001.zip › S1 Data/train/皖A151P8.jpg]

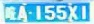

Supplement: S1 Data — (ZIP) [file pone.0339649.s001.zip › S1 Data/train/皖A155X1.jpg]

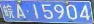

Supplement: S1 Data — (ZIP) [file pone.0339649.s001.zip › S1 Data/train/皖A15904.jpg]

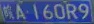

Supplement: S1 Data — (ZIP) [file pone.0339649.s001.zip › S1 Data/train/皖A160R9.jpg]

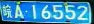

Supplement: S1 Data — (ZIP) [file pone.0339649.s001.zip › S1 Data/train/皖A16552.jpg]

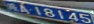

Supplement: S1 Data — (ZIP) [file pone.0339649.s001.zip › S1 Data/train/皖A18145.jpg]

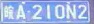

Supplement: S1 Data — (ZIP) [file pone.0339649.s001.zip › S1 Data/train/皖A210N2.jpg]

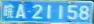

Supplement: S1 Data — (ZIP) [file pone.0339649.s001.zip › S1 Data/train/皖A21158.jpg]

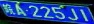

Supplement: S1 Data — (ZIP) [file pone.0339649.s001.zip › S1 Data/train/皖A225J1.jpg]

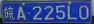

Supplement: S1 Data — (ZIP) [file pone.0339649.s001.zip › S1 Data/train/皖A225L0.jpg]

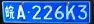

Supplement: S1 Data — (ZIP) [file pone.0339649.s001.zip › S1 Data/train/皖A226K3.jpg]

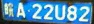

Supplement: S1 Data — (ZIP) [file pone.0339649.s001.zip › S1 Data/train/皖A22U82.jpg]

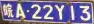

Supplement: S1 Data — (ZIP) [file pone.0339649.s001.zip › S1 Data/train/皖A22Y13.jpg]

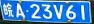

Supplement: S1 Data — (ZIP) [file pone.0339649.s001.zip › S1 Data/train/皖A23V61.jpg]

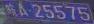

Supplement: S1 Data — (ZIP) [file pone.0339649.s001.zip › S1 Data/train/皖A25575.jpg]

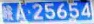

Supplement: S1 Data — (ZIP) [file pone.0339649.s001.zip › S1 Data/train/皖A25654.jpg]

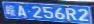

Supplement: S1 Data — (ZIP) [file pone.0339649.s001.zip › S1 Data/train/皖A256R2.jpg]

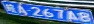

Supplement: S1 Data — (ZIP) [file pone.0339649.s001.zip › S1 Data/train/皖A267A8.jpg]

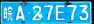

Supplement: S1 Data — (ZIP) [file pone.0339649.s001.zip › S1 Data/train/皖A27E73.jpg]

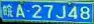

Supplement: S1 Data — (ZIP) [file pone.0339649.s001.zip › S1 Data/train/皖A27J48.jpg]

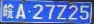

Supplement: S1 Data — (ZIP) [file pone.0339649.s001.zip › S1 Data/train/皖A27Z25.jpg]

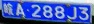

Supplement: S1 Data — (ZIP) [file pone.0339649.s001.zip › S1 Data/train/皖A288J3.jpg]

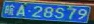

Supplement: S1 Data — (ZIP) [file pone.0339649.s001.zip › S1 Data/train/皖A28S79.jpg]

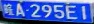

Supplement: S1 Data — (ZIP) [file pone.0339649.s001.zip › S1 Data/train/皖A295E1.jpg]

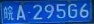

Supplement: S1 Data — (ZIP) [file pone.0339649.s001.zip › S1 Data/train/皖A295G6.jpg]

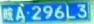

Supplement: S1 Data — (ZIP) [file pone.0339649.s001.zip › S1 Data/train/皖A296L3.jpg]

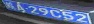

Supplement: S1 Data — (ZIP) [file pone.0339649.s001.zip › S1 Data/train/皖A29C52.jpg]

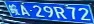

Supplement: S1 Data — (ZIP) [file pone.0339649.s001.zip › S1 Data/train/皖A29R72.jpg]

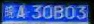

Supplement: S1 Data — (ZIP) [file pone.0339649.s001.zip › S1 Data/train/皖A30B03.jpg]

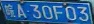

Supplement: S1 Data — (ZIP) [file pone.0339649.s001.zip › S1 Data/train/皖A30F03.jpg]

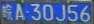

Supplement: S1 Data — (ZIP) [file pone.0339649.s001.zip › S1 Data/train/皖A30J56.jpg]

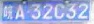

Supplement: S1 Data — (ZIP) [file pone.0339649.s001.zip › S1 Data/train/皖A32C32.jpg]

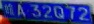

Supplement: S1 Data — (ZIP) [file pone.0339649.s001.zip › S1 Data/train/皖A32Q72.jpg]

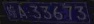

Supplement: S1 Data — (ZIP) [file pone.0339649.s001.zip › S1 Data/train/皖A33673.jpg]

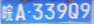

Supplement: S1 Data — (ZIP) [file pone.0339649.s001.zip › S1 Data/train/皖A339Q9.jpg]

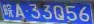

Supplement: S1 Data — (ZIP) [file pone.0339649.s001.zip › S1 Data/train/皖A33Q56.jpg]

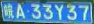

Supplement: S1 Data — (ZIP) [file pone.0339649.s001.zip › S1 Data/train/皖A33Y37.jpg]

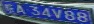

Supplement: S1 Data — (ZIP) [file pone.0339649.s001.zip › S1 Data/train/皖A34V88.jpg]

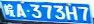

Supplement: S1 Data — (ZIP) [file pone.0339649.s001.zip › S1 Data/train/皖A373H7.jpg]

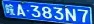

Supplement: S1 Data — (ZIP) [file pone.0339649.s001.zip › S1 Data/train/皖A383N7.jpg]

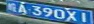

Supplement: S1 Data — (ZIP) [file pone.0339649.s001.zip › S1 Data/train/皖A390X1.jpg]

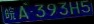

Supplement: S1 Data — (ZIP) [file pone.0339649.s001.zip › S1 Data/train/皖A393H5.jpg]

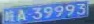

Supplement: S1 Data — (ZIP) [file pone.0339649.s001.zip › S1 Data/train/皖A39993.jpg]

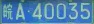

Supplement: S1 Data — (ZIP) [file pone.0339649.s001.zip › S1 Data/train/皖A40035.jpg]

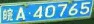

Supplement: S1 Data — (ZIP) [file pone.0339649.s001.zip › S1 Data/train/皖A40765.jpg]

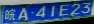

Supplement: S1 Data — (ZIP) [file pone.0339649.s001.zip › S1 Data/train/皖A41E23.jpg]

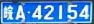

Supplement: S1 Data — (ZIP) [file pone.0339649.s001.zip › S1 Data/train/皖A42154.jpg]

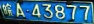

Supplement: S1 Data — (ZIP) [file pone.0339649.s001.zip › S1 Data/train/皖A43877.jpg]

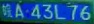

Supplement: S1 Data — (ZIP) [file pone.0339649.s001.zip › S1 Data/train/皖A43L76.jpg]

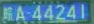

Supplement: S1 Data — (ZIP) [file pone.0339649.s001.zip › S1 Data/train/皖A44241.jpg]

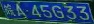

Supplement: S1 Data — (ZIP) [file pone.0339649.s001.zip › S1 Data/train/皖A45G33.jpg]

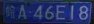

Supplement: S1 Data — (ZIP) [file pone.0339649.s001.zip › S1 Data/train/皖A46E18.jpg]

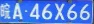

Supplement: S1 Data — (ZIP) [file pone.0339649.s001.zip › S1 Data/train/皖A46X66.jpg]

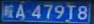

Supplement: S1 Data — (ZIP) [file pone.0339649.s001.zip › S1 Data/train/皖A479T8.jpg]

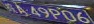

Supplement: S1 Data — (ZIP) [file pone.0339649.s001.zip › S1 Data/train/皖A49P06.jpg]

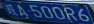

Supplement: S1 Data — (ZIP) [file pone.0339649.s001.zip › S1 Data/train/皖A500R6.jpg]

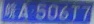

Supplement: S1 Data — (ZIP) [file pone.0339649.s001.zip › S1 Data/train/皖A506T7.jpg]

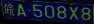

Supplement: S1 Data — (ZIP) [file pone.0339649.s001.zip › S1 Data/train/皖A508X8.jpg]

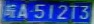

Supplement: S1 Data — (ZIP) [file pone.0339649.s001.zip › S1 Data/train/皖A512T3.jpg]

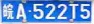

Supplement: S1 Data — (ZIP) [file pone.0339649.s001.zip › S1 Data/train/皖A522T5.jpg]

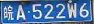

Supplement: S1 Data — (ZIP) [file pone.0339649.s001.zip › S1 Data/train/皖A522W6.jpg]

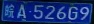

Supplement: S1 Data — (ZIP) [file pone.0339649.s001.zip › S1 Data/train/皖A526G9.jpg]

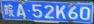

Supplement: S1 Data — (ZIP) [file pone.0339649.s001.zip › S1 Data/train/皖A52K60.jpg]

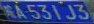

Supplement: S1 Data — (ZIP) [file pone.0339649.s001.zip › S1 Data/train/皖A531J3.jpg]

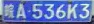

Supplement: S1 Data — (ZIP) [file pone.0339649.s001.zip › S1 Data/train/皖A536K3.jpg]

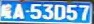

Supplement: S1 Data — (ZIP) [file pone.0339649.s001.zip › S1 Data/train/皖A53D57.jpg]

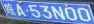

Supplement: S1 Data — (ZIP) [file pone.0339649.s001.zip › S1 Data/train/皖A53N00.jpg]

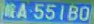

Supplement: S1 Data — (ZIP) [file pone.0339649.s001.zip › S1 Data/train/皖A551B0.jpg]

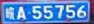

Supplement: S1 Data — (ZIP) [file pone.0339649.s001.zip › S1 Data/train/皖A55756.jpg]

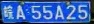

Supplement: S1 Data — (ZIP) [file pone.0339649.s001.zip › S1 Data/train/皖A55A25.jpg]

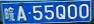

Supplement: S1 Data — (ZIP) [file pone.0339649.s001.zip › S1 Data/train/皖A55Q00.jpg]

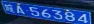

Supplement: S1 Data — (ZIP) [file pone.0339649.s001.zip › S1 Data/train/皖A56384.jpg]

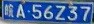

Supplement: S1 Data — (ZIP) [file pone.0339649.s001.zip › S1 Data/train/皖A56Z37.jpg]

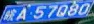

Supplement: S1 Data — (ZIP) [file pone.0339649.s001.zip › S1 Data/train/皖A57Q80.jpg]

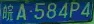

Supplement: S1 Data — (ZIP) [file pone.0339649.s001.zip › S1 Data/train/皖A584P4.jpg]

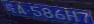

Supplement: S1 Data — (ZIP) [file pone.0339649.s001.zip › S1 Data/train/皖A586H7.jpg]

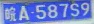

Supplement: S1 Data — (ZIP) [file pone.0339649.s001.zip › S1 Data/train/皖A587S9.jpg]

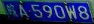

Supplement: S1 Data — (ZIP) [file pone.0339649.s001.zip › S1 Data/train/皖A590W8.jpg]

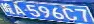

Supplement: S1 Data — (ZIP) [file pone.0339649.s001.zip › S1 Data/train/皖A596C7.jpg]

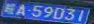

Supplement: S1 Data — (ZIP) [file pone.0339649.s001.zip › S1 Data/train/皖A59D31.jpg]

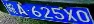

Supplement: S1 Data — (ZIP) [file pone.0339649.s001.zip › S1 Data/train/皖A625X0.jpg]

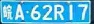

Supplement: S1 Data — (ZIP) [file pone.0339649.s001.zip › S1 Data/train/皖A62R17.jpg]

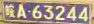

Supplement: S1 Data — (ZIP) [file pone.0339649.s001.zip › S1 Data/train/皖A63244.jpg]

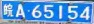

Supplement: S1 Data — (ZIP) [file pone.0339649.s001.zip › S1 Data/train/皖A65154.jpg]

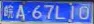

Supplement: S1 Data — (ZIP) [file pone.0339649.s001.zip › S1 Data/train/皖A67L10.jpg]

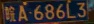

Supplement: S1 Data — (ZIP) [file pone.0339649.s001.zip › S1 Data/train/皖A686L3.jpg]

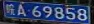

Supplement: S1 Data — (ZIP) [file pone.0339649.s001.zip › S1 Data/train/皖A69858.jpg]

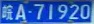

Supplement: S1 Data — (ZIP) [file pone.0339649.s001.zip › S1 Data/train/皖A71920.jpg]

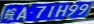

Supplement: S1 Data — (ZIP) [file pone.0339649.s001.zip › S1 Data/train/皖A71H99.jpg]

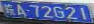

Supplement: S1 Data — (ZIP) [file pone.0339649.s001.zip › S1 Data/train/皖A72G21.jpg]
